# Editorial: The oral-gut axis: from ectopic colonization to within-host evolution of oral bacteria

**DOI:** 10.3389/fcimb.2024.1378237

**Published:** 2024-02-13

**Authors:** Mohamed M. H. Abdelbary, Rehab Z. Abdallah, Hee Sam Na

**Affiliations:** ^1^ Division of Nosocomial Pathogens and Antibiotic Resistances, Department of Infectious Diseases, Robert Koch Institute, Wernigerode, Germany; ^2^ Division of Oral Microbiology and Immunology, Department of Operative Dentistry, Periodontology and Preventive Dentistry, Rheinisch-Westfälische Technische Hochschule (RWTH) University Hospital, Aachen, Germany; ^3^ Bioinformatics Department, College of Biotechnology, Mirs University for Science and Technology, Cairo, Egypt; ^4^ Biology Department, School of Sciences and Engineering, The American University in Cairo, New Cairo, Egypt; ^5^ Department of Oral Microbiology, School of Dentistry, Pusan National University, Yangsan, Republic of Korea

**Keywords:** oral microbiome, gut microbiome, oral-gut axis, ectopic colonization, inflammatory bowel disease, colorectal cancer, Alzheimer’s disease

The human body encompasses intricate microbial communities, exhibiting non-uniform distribution across distinct body regions, each characterized by unique taxa composition and abundance. These microbial habitats are characterized by interconnectivity rather than isolation, manifesting as a complex and intertwined landscape within each individual. A noteworthy example is the gastrointestinal tract, spanning from the oral cavity to the anus, housing diverse bacterial communities.

Prior investigations have extensively characterized the composition and dynamics of human bacterial communities in the oral cavity and intestine, elucidating their indispensable roles in maintaining health and contributing to disease states. Specifically, the presence of particular oral species belonging to genera such as *Streptococcus*, *Campylobacter*, and *Fusobacterium*, within the gut microbiota has demonstrated a significant association with pathological conditions such as inflammatory bowel disease (IBD). Despite these advancements, the fundamental role of oral bacteria in the colonization of the human gut remains incompletely understood. Abdelbary et al. addressed this gap by studying the salivary and fecal microbiome in 14 IBD patients compared to 12 healthy controls and identified common bacterial taxa among both oral-gut niches. The study revealed a significant shift in the overall microbial composition of saliva in IBD patients compared to controls. At the genus level, *Veillonella* and *Prevotella* were notably more abundant in IBD patients than in the control group. Remarkably, *Prevotella salivae* emerged as a distinct oral species significantly associated with IBD. In the fecal microbiome, IBD patients exhibited a significantly higher abundance of pathogenic bacteria, including *Clostridium sensu stricto* 1 and *Escherichia-Shigella*, along with upregulation of certain metabolic pathways such as bacterial invasion of epithelial cells. *Streptococcus* was the only common genus detected in both the salivary and fecal microbiome, emphasizing the oral-gut axis. Furthering the exploration, Hammad et al. delved deeper into the salivary microbiomes of IBD patients and healthy controls using several approaches. Isolation efforts yielded significant numbers of strains, with *Veillonella* spp. and *Prevotella* spp. being more prevalent in IBD patients. RT-qPCR findings aligned with previous 16S rRNA amplicon sequencing data, confirming the higher abundance of these bacterial groups, including *P. salivae*, in the saliva of IBD patients compared to healthy controls. In contrast to RT-qPCR, 16S rRNA amplicon sequencing data allowed for a more comprehensive assessment of absolute abundance of all three bacterial groups in both IBD patients and controls.

The crosstalk between oral bacteria and immune system’s response was also a key focus of this Research Topic. For instance, Kim et al. investigated the interplay between the host’s adaptive immune system and *Fusobacterium nucleatum* (FN) in patients with colorectal cancer (CRC). FN abundance was associated with poorer disease-free and overall survival. FN infection correlated with T cell depletion, an increase in exhausted CD8^+^ and FoxP3^+^ regulatory T cells. Furthermore, FN-positive tumors exhibited higher levels of immune checkpoint inhibitory receptors in tumor-infiltrating lymphocytes, underscoring the direct impact of FN on T cell responses and immune checkpoint receptors. In another study by Aguirre-Garcia et al., the cytokine profile and characteristics of the oral and gut microbiota in Mexican patients with obesity-related hypertension were explored. The study revealed a significant enrichment of *Kluyvera* in the oral microbiota of obese individuals compared to those who were overweight. Moreover, a negative correlation emerged between proinflammatory cytokines (interleukin-6, interleukin-1β, tumor necrosis factor, and interferon-γ) and specific genera of gut bacteria (*Bacteroides*, *Alloprevotella*, and *Veillonella*) in overweight individuals, suggesting a potential link between gut dysbiosis and inflammation in the context of weight status.

The oral microbiome not only correlates with disease conditions but also with a healthy state, as highlighted by two studies in this Research Topic. Lim et al. conducted a comprehensive analysis examining the associations between the oral microbiome and 15 metabolic along with 19 complete blood count (CBC)-based markers, in a cohort of 692 healthy Korean individuals. The study revealed a significant link between the richness of the oral microbiome and four CBC markers, coupled with one metabolic marker. Notably, compositional variations in the oral microbiome were significantly influenced by fasting glucose, fasting insulin, white blood cell count, and total leukocyte count. The study identified specific microbial genera, including *Treponema*, *TG5*, and *Tannerella*, exhibiting relative abundances associated with these biomarkers, suggesting a bidirectional relationship between the oral microbiome health conditions. In a separate study by Lee et al., researchers investigated the gut and oral microbiota of 83 Korean women to delineate the core microbiomes of the intestine and oral cavity. The research aimed to explore their correlation and predict metabolic pathways based on enterotypes and orotypes. The study unveiled three enterotypes for gut bacteria and three orotypes for oral bacteria, each associated with distinct predicted metabolic pathways. *Eubacterium_g11*, *Actinomyces*, *Atopobium*, and *Enterococcus* displayed significant positive correlations in both gut and oral abundance. These bacteria were classified as type 3 in orotype and type 2 in enterotype, underlining their relevance in the crosstalk between the gut and oral microbiomes.

On the other hand, Weber et al. explored the potential relevance of the oral microbiota in Alzheimer’s disease (AD) by discussing evidence of observed shifts in the oral microbiome, especially *Porphyromonas gingivalis*, in AD patients. The review emphasizes the challenges of reverse causality, altered oral hygiene patterns due to dementia onset, and limitations of cross-sectional study designs. The link between the oral microbiota and AD may involve the presence of oral-associated bacteria in the brain or the translocation of inflammation-inducing bacterial material, such as gingipain or lipopolysaccharide. These mechanisms align with increased detection of microbial DNA and proteins, including those of *P. gingivalis*, in the brains of AD patients. The authors proposed lactoferrin as a potential link between the oral microbiota and host inflammatory conditions. However, the connections discussed remain hypothetical, with clinical studies showing inconsistency in microbiome features associated with AD.

Taken together, these studies contribute valuable insights into the intricate interplay between oral and gut microbiota, shedding light on their roles in health and systemic diseases. We express gratitude to all authors and reviewers for their contributions to this Research Topic, and we hope readers find enjoyment in the published studies.

## Author contributions

MA: Conceptualization, Project administration, Writing – original draft, Writing – review & editing. RA: Writing – review & editing. HN: Writing – review & editing.

